# An exercise prescription for patients with lung cancer improves the quality of life, depression, and anxiety

**DOI:** 10.3389/fpubh.2022.1050471

**Published:** 2022-11-17

**Authors:** Juntian Lei, Jianyu Yang, Lei Dong, Jilai Xu, Jing Chen, Xiao Hou, Zhenmin Bai

**Affiliations:** ^1^School of Sports Medicine and Rehabilitation, Beijing Sport University, Beijing, China; ^2^China-Japan Friendship Hospital, Beijing, China; ^3^School of Sport Science, Beijing Sport University, Beijing, China

**Keywords:** exercise prescription, lung cancer, depression, anxiety, quality of life

## Abstract

**Introduction:**

Lung cancer has the highest rates of morbidity and mortality among all cancers. Patients with lung cancer inevitably confront psychosocial discomforts and progressively experience depression and anxiety that potentially impact the clinical outcomes [e.g., quality of life (QoL)]. These mental disorders in patients with lung cancer may effectively be alleviated with prescribed Chinese traditional mind-body exercises. This study aimed to determine the effect of the exercise prescription containing Chinese traditional mind-body exercise on QoL, depression, and anxiety in patients with lung cancer.

**Methods:**

In this study, 52 patients with non-small cell lung cancer (NSCLC) recruited from the China-Japan Friendship Hospital were divided into two groups, namely, the experimental group (*N* = 26) and the control group (*N* = 26). The experimental group was treated with an 8-week exercise prescription containing aerobic and resistance training. The control group received the usual care during the study period. The QoL, depression, and anxiety were separately investigated using EORTC QLQ-C30, EORTC QLQ-LC13, the Self-Rating Depression Scale (SDS), and the Self-Rating Anxiety Scale (SAS) at baseline and post-intervention. The scores of questionnaires were analyzed using the paired sample and independent sample t-tests to explore the intragroup and intergroup differences, respectively.

**Results:**

The EORTC QLQ-C30 scores for physical functioning, role functioning, emotional functioning, and global QoL in the experimental group at post-intervention were significantly higher than those at baseline. The EORTC QLQ-C30 scores for fatigue, pain, dyspnea, and insomnia in the experimental group at post-intervention were significantly lower than those at the baseline. The SDS scores (baseline: 57.74 ± 8.77 vs. post-intervention: 51.42 ± 7.31, *p* < 0.05) and the SAS scores (baseline: 56.63 ± 9.39 vs. post-intervention: 49.16 ± 7.83, *p* < 0.05) in the experimental group at post-intervention were significantly lower than those at baseline.

**Conclusions:**

The 8-week exercise prescription containing moderate-intensity Baduanjin (5 days per week) can effectively alleviate QoL, depression, and anxiety in patients with NSCLC. Our exercise prescription is an effective supportive treatment for lung cancer patients with depression and anxiety.

**Clinical trial registration:**

Chinese Clinical Trial Registry (ChiCTR1900025121).

## Introduction

Lung cancer has the highest rates of morbidity and mortality among all cancer types in the world (1–3). In 2003–2009, the 5-year relative survival rate for patients diagnosed with lung cancer was only 18.2% (1). In China, lung cancer is the most common type of cancer and the leading cause of death (3, 4). With the sharp rise in smoking rates in China, the incidence of lung cancer may still be increasing (5–7). According to the statistics of the World Health Organization, the annual lung cancer mortality rate in China may reach 1 million by 2025.

Due to the disease deterioration and side effects of treatment, patients with lung cancer not only experience physiological discomforts, including pain, weight loss, and dyspnea (8), but also inevitably confront psychosocial problems, which progressively develop into depression and anxiety (9–11). For example, Linden et al. (12) found that anxiety and depression levels vary widely in patients diagnosed with different cancers, and the depression level in patients with lung cancer is the highest. A 6-month prospective study found that 29% of patients with lung cancer have depression after thoracotomy, leading to a poorer emotional QoL (11). Moreover, a recent study also showed that patients with lung cancer who suffer from emotional problems have a lower QoL and heavier symptom burdens than those who report no emotional problems (13). Thus, the negative effects of depression and anxiety should be seriously considered during the implementation of treatment for patients with lung cancer.

Physical exercise has been recognized as a feasible and effective way to improve clinical outcomes in patients with lung cancer after surgery (14). Although different combinations of exercise types (e.g., aerobic exercise, resistance training, and high-intensity interval training) have been proven to improve different physical capacities, such as peak rate of oxygen uptake (VO_2_peak), respiratory muscle strength, 6-min walking test (6MWT) distance, fatigue, and health-related QoL (15–18) of the patients with lung cancer, the effect of exercise interventions on alleviating depression and anxiety in patients with lung cancer remains unclear (19, 20). Only a few studies considered the impact of exercise on the emotional problems of patients with lung cancer, and most of their exercise prescriptions failed to alleviate depression and anxiety (21–23).

Baduanjin, a moderate-intensity aerobic exercise derived from Tai Chi/Qigong, contains eight serial movements designed for people of all ages to maintain both mental and physical health. Because of its simplicity, cost-effectiveness, and utility, Baduanjin has been popular in China for over 800 years. Furthermore, it can be considered a Chinese traditional mind-body exercise involving body relaxation, mental imagery, and mindfulness (24, 25). These mental processes may potentially improve cognition, attention, and mood by retaining a pattern of activation of brain regions and connectivity of brain networks (26). It has been demonstrated that Baduanjin shows effectiveness at both physical and mental levels on mental disorders and some other specific diseases, such as depression, anxiety (27), insomnia (28), musculoskeletal pain (29), chronic obstructive pulmonary disease (30, 31), and cancers (32). However, these studies mainly focused on patients with breast cancer (33, 34) rather than those with lung cancer. There is still no scientific evidence supporting the effect of Baduanjin exercise prescriptions on the mental health of patients with lung cancer.

Given that depression and anxiety can negatively impact the clinical outcomes of patients with lung cancer, there is an urgent need for effective and targeted exercise prescriptions for patients with lung cancer who have mental disorders. The aim of this study was to determine the effect of the exercise prescription with Baduanjin on QoL, depression, and anxiety in patients with lung cancer. We hypothesized that our exercise prescription with Baduanjin had a significant benefit on the QoL, depression, and anxiety in patients with lung cancer.

## Methods

### Study design and participants

This was a parallel, pseudorandomized, controlled, and single-center trial conducted at the China-Japan Friendship Hospital in Beijing, China. A convenience sample was assigned to the control group (CG) and the experimental group (EG). The EG was treated with an 8-week exercise prescription containing Baduanjin. The CG received the usual care during the study period. The recruitment started from June 2019 to December 2019.

The inclusion criteria for participants were as follows: (1) individuals aged between 18 and 75 years; (2) those with histologically confirmed stage I-IIIB non-small cell lung cancer (NSCLC) and in a medically stable condition for the last 8 weeks; (3) those with the Eastern Cooperative Oncology Group Performance Status (ECOG PS) scores ≤2; (4) those with a life expectancy of ≥12 weeks; (5) those who performed <150 min of moderate-intensity exercise per week; (6) those who were suggested no further medical examination by ACSM's Pre-activity Screening Questionnaire (PASQ); (7) those who were able to complete the exercise prescription with normal neuromuscular and cognitive functions; and (8) those who for completed all definitive therapy (i.e. surgery, radiation, or chemotherapy) more than 1 week without severe complications related to these therapies.

The exclusion criteria for participants were as follows: (1) individuals with symptomatic spinal cord compression or brain metastasis; (2) aberrant cardiac function or any cardiac case history; (3) severe systemic disease; (4) autoimmune disease; (5) cancer-related active bleeding within 3 months; (6) psychiatric disorder history or contraindications for exercise (i.e., severe osteoporosis and musculoskeletal disorders); (7) positive blood or urine pregnancy tests within 3 days before the intervention; (8) disease related to cortisol increment; (9) individuals scheduled to have surgery or chemotherapy within 12 weeks; and (10) in situations where the doctor recommended not to be involved in any form of exercise.

The participants were considered dropout subjects when they (1) were unable to continue the experiment because of the deteriorated disease progression; (2) declined to continue; (3) had an adherence rate of intervention of ≤80%; (4) died; and (5) had sports injuries during the experiment.

The research was approved by the Sports Science Experiment Ethics Committee of Beijing Sport University (2019064H).

### Procedure

The patients from the respiratory and critical illness medicine department and internal medicine department of China-Japan Friendship Hospital were screened according to the inclusion and exclusion criteria. Eligible patients were informed about the study procedure and invited to participate in this study. All participants provided informed consent and then underwent the baseline assessment. Patients were assigned in the order of their visits. The first 27 patients were assigned to the CG, whereas the following assigned patients were assigned to the EG. The EG was treated with our 8-week exercise prescription, and the CG received the usual care. The European Organization for Research and Treatment of Cancer Quality of Life Questionnaire (EORTC QLQ-C30 and EORTC QLQ-LC13), the Self-Rating Depression Scale (SDS), and the Self-Rating Anxiety Scale (SAS) were sent to participants by social media after the intervention. For ethical reasons, the participants in CG were given systematic exercise guidance after the experiment.

### Intervention

The patients in the EG received detailed movement instructions for the exercise prescriptions from therapists before the intervention. In addition, the brochures and online videos of the movement instructions were distributed to patients for review. In the first 2 weeks, the patients in the EG performed the exercise prescription in the hospital and were supervised by the therapists. In the last 6 weeks, the patients in the EG were asked to exercise at home and to record their training details on their record cards. The therapist checked the training progress online two times a week *via* video call or other social media.

**Figure 1:**
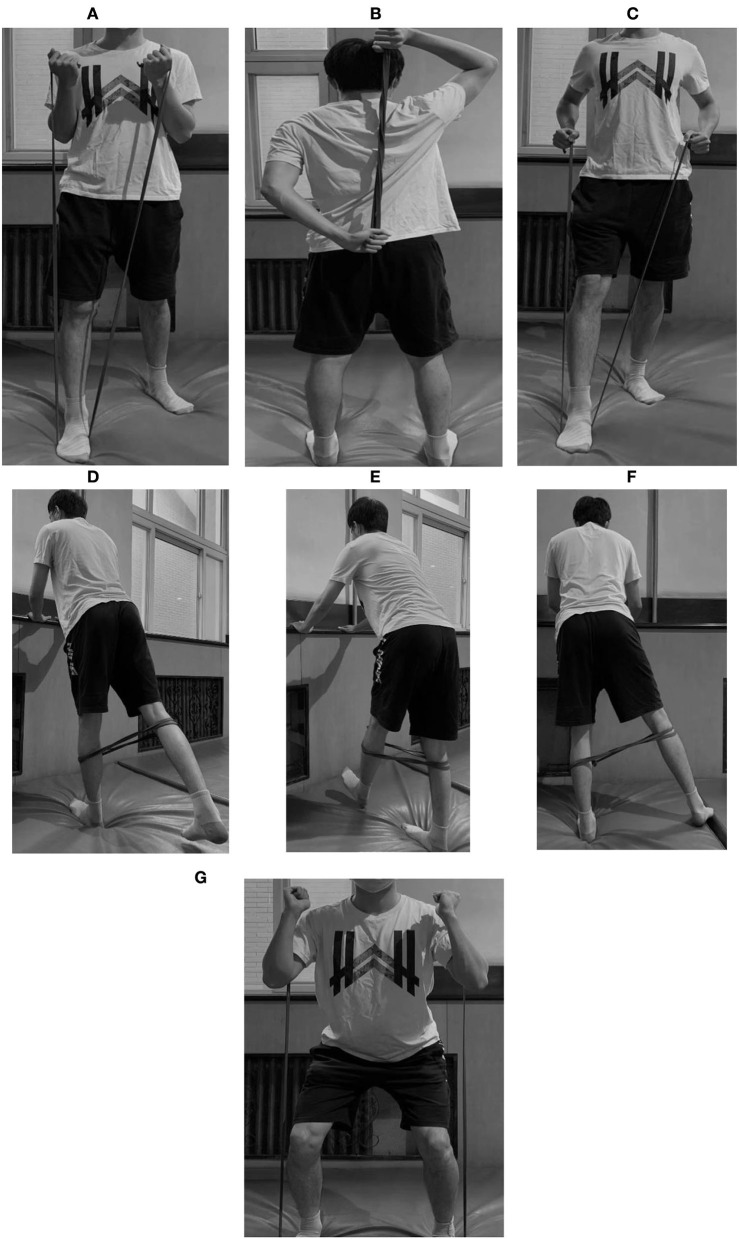
Movements in elastic band training. **(A)** Standing elbow flexion. **(B)** Standing elbow extension. **(C)** Standing rowing. **(D)** Standing hip extension. **(E)** Standing hip flexion. **(F)** Standing hip abduction. **(G)** Squat.

There were two different training schemes in the exercise prescription. The first scheme focused on Baduanjin practice. It contained a 26-min Baduanjin session, a 10-min warm-up session, and a 5-min warm-down session. The second scheme involved resistance training and Baduanjin. It contained a 12-min Baduanjin session, a 23-min elastic band training session, a 10-min warm-up session, and a 5-min warm-down session. Both schemes lasted ~50 min each time. The intervention frequency gradually increased from three times per week to five times per week. In addition, the exercise intensity gradually increased from the rest state to 65%−75% of maximum heart rate (HR_max_) for Baduanjin and 30%−50%^*^1RM for resistance training (shown in Table 1). Heart rate was monitored continuously by the heart rate chest belt (Inc. Polar, China) during the exercise. The specific movements of elastic band training are exhibited in Table 2 and Figure 1.

**Table 1:** Details of exercise prescription.

	**Type**	**Time**	**Intensity**	**Frequency**
The former 2 weeks	Baduanjin	Warm-up: 10 min Baduanjin: 26 min Warm-down: 5 min	65%−75%HR_max_	2 times/week
	Baduanjin and EBT	Warm-up: 10 min Baduanjin: 12 min EBT: 23 min Warm-down: 5 min	Baduanjin: 65%−75%HR_max_ EBT: 30%−50%*1RM, 10–12 reps/set, 3 sets	1 time/week
The latter 6 weeks	Baduanjin	Warm-up: 10 min Baduanjin: 26 min Warm-down: 5 min	65%−75%HR_max_	3 times/week
	Baduanjin and EBT	Warm-up: 10 min Baduanjin: 12 min EBT: 23 min Warm-down: 5 min	Baduanjin: 65%−75%HR_max_ EBT: 30%−50%*1RM, 10–12 reps/set, 3 sets	2 times/week

EBT, elastic band training; HRmax, maximum heart rate; reps, repetitions.

**Table 2:** Movements in elastic band training.

**Elastic band training**	
Standing elbow flexion	The patient stands in the middle of the elastic band and grabs two ends of the elastic band in each hand. Both hands are moved into an elbow flexion motion and then back to neutral
Standing elbow extension	With the elastic band grabbed in each hand, the patient puts one hand behind the back and the other hand behind the head. Cues are given to pull the elastic band apart
Standing rowing	The patient places one foot a step forward and then puts the midpoint of the elastic band under the front foot. The patient begins by holding the elastic band with both hands. Cues are given to squeeze the shoulder blades together without allowing them to shrug
Standing hip extension	With the two ends of the elastic band tied together, the patient places both legs inside the loop of the band. Keeping the knee slightly bent, and the trunk steady, the patient stabilizes on one leg. The contralateral leg is moved into a hip extension motion and then back to neutral
Standing hip flexion	With the two ends of the elastic band tied together, the patient places both legs inside the loop of the band. Keeping the knee slightly bent, and the trunk steady, the patient stabilizes on one leg. The contralateral leg is moved into a hip flexion motion and then back to neutral
Standing hip abduction	With the two ends of the elastic band tied together, the patient places both legs inside the loop of the band. Keeping the knee slightly bent, and the trunk steady, the patient stabilizes on one leg. The contralateral leg is moved into a hip abduction motion and then back to neutral
Squat	The patient stands on the elastic band and grabs the elastic band in each hand. The patient performs a squatting motion to 45 degrees of knee flexion while keeping his knees behind his feet during the exercise. The patient completes the exercise by returning to the start position

### Outcomes

Demographic and clinical characteristics of patients, including age, sex, smoking history, cancer diagnosis, and surgery condition, were collected at baseline. The primary outcomes were the scores of SDS and SAS. Both SDS and SAS cover 20 items corresponding to depression- and anxiety-related symptoms. Points 1–4 represent a few of the times, some of the times, a good part of the time, and most of the time, respectively (35, 36). The scale raw scores were multiplied by 1.25 and rounded to the nearest integer (35). The higher scores indicate a worse status of depression or anxiety. The secondary outcomes of the study were the scores of the EORTC QLQ-C30 and -LC13 subscales. The QLQ-C30 is a reliable and valid approach for evaluating the QoL of patients with cancer (37). It consists of five functional scales, three symptom scales, a global health and quality of life scale, and six single-item symptom scales. The QLQ-LC13 as a supplement to QLQ-C30 is developed to measure the cancer-related symptoms and side effects, especially in patients with lung cancer (38). The items of QLQ-C30 and QLQ-LC13 are scored on a 4-point scale. The scores of scales are linearly transformed to a 0–100 scale according to a standardized process (39). The higher scores on the functional scale indicate a better functional status, whereas the higher scores on the symptom scale indicate a worse status of the symptom.

### Sample size

The sample size was calculated with an effect size of 0.6, a power of 0.8, and a significance level of 0.05. Considering the dropout rate of 30% in patients with lung cancer, 27 individuals were planned for each group.

### Statistical analysis

All data were extracted using the SPSS software for statistical analysis. Demographic and clinical information was described by frequencies. The scores of questionnaires and scales were reported as mean and standard deviation (mean ± SD). The chi-square test was performed in demographic and clinical information for the detection of differences between the groups at baseline. The paired sample t-test was used to analyze the differences between baseline and post-intervention. In addition, the independent sample t-test was used to analyze the differences between the CG and the EG. The level of statistical significance was set at a *p*-value < 0.05.

## Results

**Figure 2:**
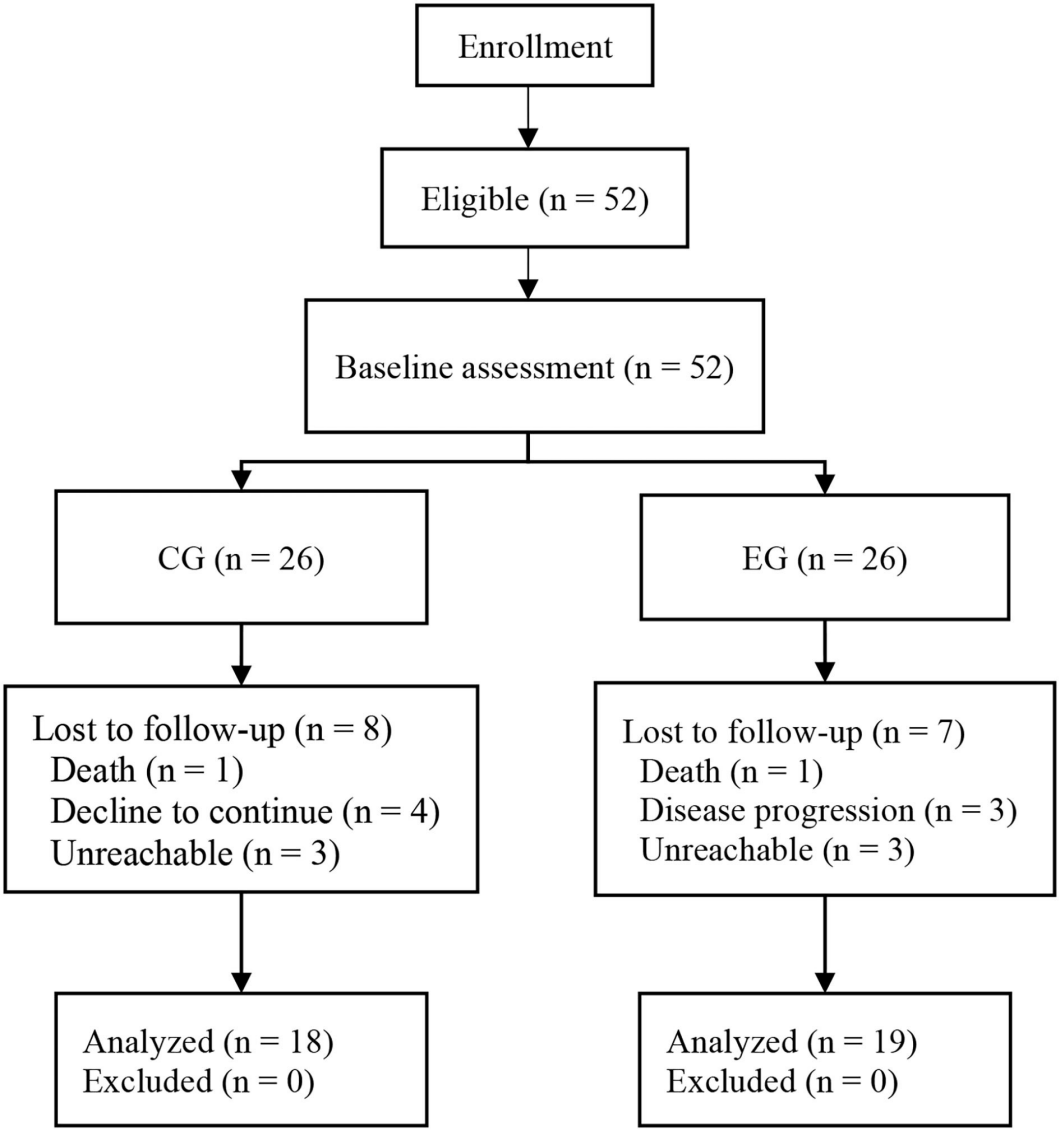
Flowchart of the study.

A total of 52 participants met the inclusion criteria of this study. In the CG, eight of the 26 participants dropped out during the intervention (one died, four declined to continue, and three were unreachable). In the EG, seven of the 26 participants dropped out during the intervention (one died, three dropped out because of deteriorated disease progression, and three were unreachable). It led to an overall adherence rate of 71.15%. No intervention-related adverse events occurred during the study period (shown in Figure 2).

The demographic and clinical characteristics of participants are shown in Table 3. Most participants were men (63.5%) and were diagnosed with lung adenocarcinoma (48.1%). Participants in the CG were aged 58.03 ± 7.71 years, and those in the EG were aged 56.04 ± 11.67 years. More than half of the participants (63.5%) did not require surgery. There was no significant difference in demographic and clinical characteristics between the CG and the EG.

**Table 3:** The demographic and clinical characteristics of participants.

	**CG (*n* = 26)**	**EG (*n* = 26)**	***p*-Value**
Age (years)	58.03 ± 7.71	56.04 ± 11.67	0.469
**Sex (mean, %)**			
Men	16 (61.5%)	17 (65.4%)	0.773
Women	10 (38.5%)	9 (34.6%)	
**Smoking history**			
Yes	14 (53.8%)	16 (61.5%)	0.266
No	12 (45.2%)	10 (38.5%)	
**Surgery**			
Yes	8 (30.8%)	11 (42.3%)	0.388
No	18 (69.2%)	15 (57.7%)	
**Cancer diagnosis**			
Adenocarcinoma	12 (46.2%)	13 (50.0%)	0.852
Squamous carcinoma	12 (46.2%)	10 (38.5%)	
Large cell carcinoma	2 (7.7%)	3 (11.5%)	

*p*-Values are based on the chi-square test for categorical variables and the t-test for continuous variables.

### Primary outcomes

As shown in Table 4, both SDS scores and SAS scores in the EG at post-intervention (SDS: 51.42 ± 7.31; SAS: 49.16 ± 7.83) were significantly lower than those at baseline (SDS: 57.74 ± 8.77, *p* < 0.05; SAS: 56.63 ± 9.39, *p* < 0.05). In addition, the SDS and SAS scores of the EG at post-intervention (SDS: 51.42 ± 7.31; SAS: 49.16 ± 7.83) were also lower than those in the CG at post-intervention (SDS: 56.94 ± 8.54, *p* < 0.05; SAS: 55.33 ± 8.69, *p* < 0.05).

**Table 4:** The SDS and SAS scores of the CG and the EG.

**Scale**	**CG (*****n*** = **18)**		**EG (*****n*** = **19)**	
	**Baseline**	**Post-intervention**	**Baseline**	**Post-intervention**
SDS	56.56 ± 8.19	56.94 ± 8.54	57.74 ± 8.77	51.42 ± 7.31^*^^#^
SAS	54.89 ± 8.71	55.33 ± 8.69	56.63 ± 9.39	49.16 ± 7.83^*^^#^

* Indicates a significant difference (*p* < 0.05) between the baseline and post-intervention.

# Indicates a significant difference (*p* < 0.05) between the CG and the EG.

### Secondary outcomes

Table 5 shows the total QLQ-C30 scores of patients with NSCLC at baseline and post-intervention. As for functional scales in the EG, there were significant differences in the QLQ-C30 scores of physical functioning (baseline: 67.67 ± 15.18 vs. post-intervention: 90.00 ± 7.95, *p* < 0.01), role functioning (baseline: 66.67 ± 27.57, vs. post-intervention: 87.50 ± 15.17, *p* < 0.01), emotional functioning (baseline: 72.08 ± 21.68 vs. post-intervention: 82.92 ± 15.87, *p* < 0.01), and global QoL (baseline: 26.67 ± 17.85 vs. post-intervention: 46.67 ± 14.15, *p* < 0.01). However, no significant difference was found in the scores of social functioning and cognitive functioning. As for symptom scales in the EG, there were significant differences in the QLQ-C30 scores of fatigue (baseline: 33.33 ± 23.36 vs. post-intervention: 18.89 ± 13.05, *p* < 0.01), pain (baseline: 30.00 ± 32.26 vs. post-intervention: 11.67 ± 16.31, *p* < 0.01), dyspnea (baseline: 31.67 ± 27.52 vs. post-intervention: 20.00 ± 16.75, *p* < 0.01), and insomnia (baseline: 41.67 ± 30.35 vs. post-intervention: 21.67 ± 16.31, *p* < 0.01). However, no significant difference was found in nausea and vomiting, appetite loss, constipation, diarrhea, and financial impact.

**Table 5:** The QLQ-C30 scores of the CG and the EG.

**QLQ-C30 item**	**CG (*****n*** = **18)**		**EG (*****n*** = **19)**	
	**Baseline**	**Post-intervention**	**Baseline**	**Post-intervention**
**Functional scales**				
Physical functioning	64.82 ± 19.10	64.44 ± 18.29	67.67 ± 15.18	90.00 ± 7.95**^##^
Role functioning	60.19 ± 29.78	57.41 ± 28.71	66.67 ± 27.57	87.50 ± 15.17**^##^
Emotional functioning	60.65 ± 18.70	65.28 ± 19.01	72.08 ± 21.68	82.92 ± 15.87**^##^
Cognitive functioning	57.41 ± 21.56	60.19 ± 22.24	70.00 ± 26.27	69.17 ± 18.16
Social functioning	58.33 ± 23.00	56.48 ± 17.28	70.83 ± 24.71	69.17 ± 18.16^#^
Global quality of life	29.17 ± 14.64	29.63 ± 15.45	26.67 ± 17.85	46.67 ± 14.15**
**Symptom scales/items**				
Fatigue	43.21 ± 22.83	43.83 ± 21.04	33.33 ± 23.36	18.89 ± 13.05**^##^
Nausea and vomiting	29.63 ± 28.33	19.44 ± 23.04*	14.17 ± 18.95	9.17 ± 10.08
Pain	41.67 ± 30.92	43.52 ± 29.23	30.00 ± 32.26	11.67 ± 16.31**^##^
Dyspnea	44.44 ± 25.57	44.44 ± 25.57	31.67 ± 27.52	20.00 ± 16.75**^##^
Sleep disturbance	55.56 ± 36.16	55.56 ± 36.16	41.67 ± 30.35	21.67 ± 16.31**^##^
Appetite loss	29.63 ± 37.73	33.33 ± 37.92	25.00 ± 26.21	13.33 ± 16.75
Constipation	22.22 ± 30.25	24.07 ± 29.83	28.33 ± 29.17	20.00 ± 16.75
Diarrhea	24.07 ± 29.83	24.07 ± 29.83	21.67 ± 31.11	15.00 ± 20.16
Financial impact	51.85 ± 32.78	51.85 ± 32.78	38.33 ± 31.11	38.33 ± 31.11

The symbols * and ** indicate a significant difference (*p* < 0.05 and *p* < 0.01, respectively) between the baseline and post-intervention; The symbols ^#^ and ^*##*^ indicate a significant difference (*p* < 0.05 and *p* < 0.01, respectively) between the CG and EG.

Compared with the CG in functional scales, the EG showed higher QLQ-C30 scores of physical functioning (CG: 64.44 ± 18.29 vs. EG: 90.00 ± 7.95, *p* < 0.01), role functioning (CG: 57.41 ± 28.71 vs. EG: 87.50 ± 15.17, *p* < 0.01), emotional functioning (CG: 65.28 ± 19.01 vs. EG: 82.92 ± 15.87, *p* < 0.01), and social functioning (CG: 56.48 ± 17.28 vs. EG: 69.17 ± 18.16, *p* < 0.05). However, no significant difference was found in the scores of cognitive functioning and global QoL. Compared with the CG in symptom scales, the EG showed higher QLQ-C30 scores of fatigue (CG: 43.83 ± 21.04 vs. EG: 18.89 ± 13.05, *p* < 0.01), pain (CG: 43.52 ± 29.23 vs. EG: 11.67 ± 16.31, *p* < 0.01), dyspnea (CG: 44.44 ± 25.57 vs. EG: 20.00 ± 16.75, *p* < 0.01), and sleep disturbance (CG: 55.56 ± 36.16 vs. EG: 21.67 ± 16.31, *p* < 0.01).

In the CG, there was no significant difference in the QLQ-C30 scores of all items except nausea and vomiting (baseline: 29.63 ± 28.3 vs. post-intervention: 19.44 ± 23.04, *p* < 0.05). Table 6 shows the total QLQ-LC13 scores of patients with NSCLC at baseline and post-intervention. The QLQ-LC13 scores of dyspnea showed a significant intragroup (baseline: 8.33 ± 25.10 vs. post-intervention: 26.11 ± 14.98, *p* < 0.01) and intergroup difference (CG: 41.36 ± 22.48 vs. EG: 26.11 ± 14.98, *p* < 0.05). However, no significance was found in the other items.

**Table 6:** The QLQ-LC13 scores of the CG and the EG.

**QLQ-LC13 item**	**CG (*****n*** = **18)**		**EG (*****n*** = **19)**	
	**Baseline**	**Post-intervention**	**Baseline**	**Post-intervention**
Dyspnea	41.36 ± 26.07	41.36 ± 22.48	38.33 ± 25.10	26.11 ± 14.98^**^^#^
Coughing	35.19 ± 24.18	37.03 ± 22.55	38.33 ± 22.36	31.67 ± 17.01
Haemoptysis	16.67 ± 20.61	16.67 ± 20.61	13.33 ± 25.13	13.33 ± 25.13
Sore mouth	16.67 ± 28.58	16.67 ± 28.58	15.00 ± 22.88	13.33 ± 19.94
Dysphagia	18.52 ± 30.73	18.52 ± 30.73	18.33 ± 22.88	16.67 ± 20.23
Peripheral neuropathy	22.22 ± 28.01	24.07 ± 22.30	25.00 ± 26.21	18.33 ± 17.01
Alopecia	46.30 ± 32.62	46.30 ± 32.62	45.00 ± 29.17	45.00 ± 29.17
Pain in chest	22.22 ± 28.01	27.78 ± 32.84	16.67 ± 20.23	16.67 ± 20.23
Pain in arm or shoulder	11.11 ± 16.17	12.96 ± 16.72	15.00 ± 17.01	16.67 ± 17.10
Pain in other parts	5.56 ± 12.78	7.41 ± 14.26	3.33 ± 10.26	5.00 ± 12.21

** Indicates a significant difference (*p* < 0.01) between the baseline and post-intervention.

# Indicates a significant difference (*p* < 0.05) between the CG and EG.

## Discussion

Our results showed that our exercise prescription improved the QoL, depression, and anxiety of patients with lung cancer. To the best of our knowledge, this is the first study to investigate the effect of exercise prescriptions, including both Baduanjin and resistance training, in patients with lung cancer. Compared with previous studies, we stress that exercise intensity and volume must be sufficient for patients with lung cancer. One study reported that the exercise prescription that combined eight-movement Tai Chi, a Chinese traditional exercise similar to Baduanjin, with resistance training for patients with lung cancer improved depression and anxiety but failed to improve most aspects of QoL (40). Compared to our exercise prescription, it included a smaller volume of aerobic exercise and a lower frequency of resistance training. These insufficiencies in exercise prescription might account for the unchanged QoL. Furthermore, Chen et al. also found that a 40-min-per-session walking exercise program without resistance training failed to improve cancer-related symptoms (e.g., pain, fatigue, nausea, and sleep disturbance) (41). As their cancer-related symptoms were mild, isolated aerobic walking at a fixed intensity might be monotonous and fail to reach the necessary intensity. We believe that progressive intensity and volume are indispensable elements in an exercise prescription adapted for patients with different physical conditions. Our study recommended a comprehensive exercise prescription that combined mind-body exercises with resistance training because it might include all essential components to treat patients with physical and mental illnesses.

Considering the physical frailty of patients with cancer, the exercises prescribed for them must be safe and easy to learn (42). In our exercise prescription, Baduanjin, a low- to moderate-intensity exercise that includes only eight movements, can be learned easily by patients with worse physical functional status. Systematic reviews demonstrated that Baduanjin effectively improves the QoL and cancer-related symptoms of postoperative breast cancer patients and is unlikely to result in serious adverse events (34, 43). Moreover, the use of elastic bands can modulate the intensity of resistance training in a controlled manner with a simple change in grip width or rubber stiffness (44). Therefore, Baduanjin combined with elastic band training as an exercise prescription might be better than medical treatment. Although patients with cancer who received medical treatment (e.g., nabilone and megestrol acetate) showed higher QoL and improved cancer-related symptoms (45), these medicines may have severe side effects (46–48). Furthermore, as no adverse events were observed during the intervention, our study confirmed the safety and effectiveness of Baduanjin combined with elastic band training. Thus, we suggest that Baduanjin combined with elastic band training is a safe exercise prescription that is easier to implement and promote in patients with lung cancer.

Our results showed that the exercise prescription containing Baduanjin improved most aspects of the functional scale and most cancer-related symptoms on the QLQ-C30, including fatigue, pain, and sleep disturbance. Its effectiveness is consistent with that reported by a meta-analysis assessing the effect of Baduanjin on QoL in patients with cancer (32). The improvement in emotional function and sleep disturbance may be related to a reduction in cortisol levels. Studies showed that people with depression or sleep disturbances have higher cortisol levels (49, 50). Baduanjin may alleviate the emotional burden and sleep disturbance by reducing cortisol excretion (51). A significant improvement in dyspnea was noted on the QLQ-LC13. As a meta-analysis demonstrated that Baduanjin could effectively improve lung function in patients with chronic obstructive pulmonary disease (52), our results indicate that the benefits of Baduanjin may also apply to patients with lung cancer. Although the scores of other items (i.e., coughing, sore mouth, and peripheral neuropathy) on the QLQ-LC13 did not improve significantly, they showed a downward trend in the EG and an upward trend in the CG post-intervention. The significant improvement of these items on the QLQ-LC13 may require a larger sample size or a longer intervention period to demonstrate. Therefore, we postulate that Baduanjin is a valuable supportive treatment for patients with lung cancer suffering from the side effects of cancer treatment.

It is worth mentioning that the item “nausea and vomiting” on the QLQ-C30 improved significantly in the CG but did not change significantly in the EG. A possible explanation for this might be that patients with comparatively high “nausea and vomiting” scores received medical treatment. Thus, there might be an unexpected factor between the CG (29.63 ± 28.33) and the EG (14.17 ± 18.95), as the “nausea and vomiting” scores were distinctly different at baseline. That is, the significant improvement in the CG may be attributed to medical treatment and the improvement trend in the EG may be attributed to the effect of our exercise prescription. Thus, we suggest a future study that considers the effects of medication to determine whether exercise can improve such symptoms.

The dropout rate in this study was 28.85%, which is not high compared to other studies of patients with lung cancer (16, 23, 40). This could be attributed to the fact that the patients in the present study were older adults. Hence, they were closely cared for by family and not bothered by work. They could then spare more time to perform the exercise prescription. During the follow-up period, we found that the patients' willingness to conduct the exercise prescription was motivated by Baduanjin. In addition, record cards for training and instructive videos reportedly increase adherence (53, 54). However, the occurrence of failure to follow up should be managed. This problem might have been caused by insufficient supervision during the 6-week home exercise program. We suggest offline or online supervised training of patients to increase the adherence rate.

This study has several limitations, and its findings should be interpreted with caution. First, owing to the lack of eligible patients in the hospital, we were unable to conduct a randomized controlled trial. It was difficult to recruit a large number of patients in a short time and implement the intervention on all participants concurrently. Therefore, the patients received the intervention once they participated in the experiment. This is a pseudorandomized procedure that inevitably leads to biased outcomes. Second, due to the nature of the exercise intervention, patient blinding was impossible, and placebo effects could not be eliminated. Third, this study did not record and control the patients' drug intakes, which may have biased the outcomes. Finally, it did not include qualitative measurements such as patient feedback; therefore, comparisons with future studies will be limited.

## Conclusion

This study demonstrated that the 8-week exercise prescription containing Baduanjin could effectively improve QoL, depression, and anxiety in patients with NSCLC. Our exercise prescription is an effective supportive treatment for lung cancer patients with depression and anxiety. Baduanjin is a safe and easy-to-learn mind-body exercise that should be included in exercise prescriptions.

## Data availability statement

The raw data supporting the conclusions of this article will be made available by the authors, without undue reservation.

## Ethics statement

The studies involving human participants were reviewed and approved by the Sports Science Experiment Ethics Committee of Beijing Sport University. The patients/participants provided their written informed consent to participate in this study.

## Author contributions

ZB and JX contributed to the concept of the work. JY, JX, LD, and JC contributed to the conduction of this trial. JL and JY contributed to data analysis and the drafting of the article. All authors contributed to the article and approved the submitted version.

## Funding

This study was funded by the Fundamental Research Funds for the Central Universities of China, grant number 2019PT014.

## Conflict of interest

The authors declare that the research was conducted in the absence of any commercial or financial relationships that could be construed as a potential conflict of interest.

## Publisher's note

All claims expressed in this article are solely those of the authors and do not necessarily represent those of their affiliated organizations, or those of the publisher, the editors and the reviewers. Any product that may be evaluated in this article, or claim that may be made by its manufacturer, is not guaranteed or endorsed by the publisher.
